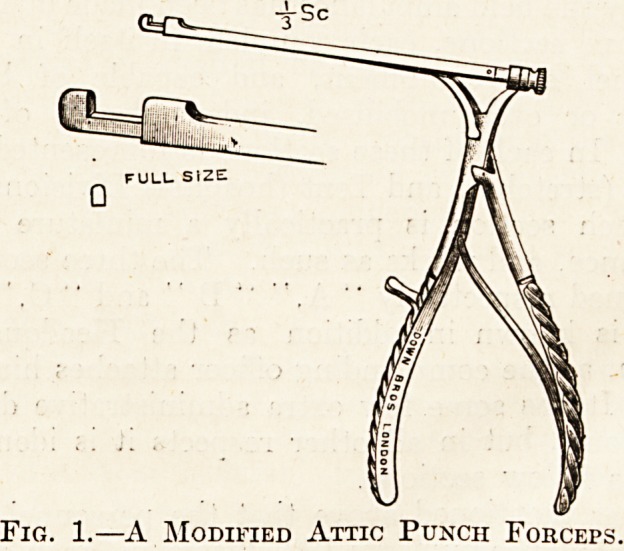# Concerning Operations for Otorrhœa

**Published:** 1907-09-21

**Authors:** 


### Otology.

### I.—CONCERNING OPERATIONS FOR OTORRHŒA.

I. Myringotomy.—In a former issue of this journal we considered myringotomy and advocated free incision of the drum head, not only for acute otitis media with severe pain and bulging membrane, but also for those cases, chiefly in infants, of chronic painless otorrhœa in which exact otoscopic examination reveals a minute nipple like projection of the membrane corresponding to a perforation so small that the thick mucus secreted is retained within the tympanum, and bulges the membrane.

II. Ossiculectomy.—In the surgical crescendo, to use a certain distinguished surgeon's expression, ossiculectomy might be regarded as the next measure in the operative scale. This operation involves the complete removal of the remains of the tympanic membrane from its attachment to the annulus tympanicus, disarticulation and removal of the malleus and also of the incus, but not of the stapes. All granulations are then curetted. Formerly the operation ended here, but seeing that better access to the attic is obtained by removal of the upper tympanic margin, which forms the outer attic wall, that additional procedure is now regarded as a necessary stage in the operation. This margin can be removed with suitable instruments without adding to the severity of the operation. Be it remembered that it is the attic which holds the bulk of the ossicles, the head and the neck of the malleus, the body of the incus with its shorter, and part of its longer crus. It is, presumably, the ossicles which are the seat of disease, in the cases for which this operation is selected.

This complete tympanic operation is especially suitable when granulations continue to grow in the tympanic cavity, and to obstruct the entrance of the antrum, in spite of treatment by other and non-operative measures. The formation of such granulations is often associated with ossicular caries, although it is not always possible to demonstrate a definite pathological lesion of the bone before its removal. Of the two, the incus is much more frequently affected than the malleus; moreover, removal of the malleus alone is rarely followed by cessation of suppuration. The operation cannot be performed if the meatus is unusually small.

*Technique.*—The patient having been prepared for the operation, is placed in the recumbent position, and anæsthetised. The auditory canal is disinfected, irrigated and dried. A good light is essential, and must be directed through a large speculum to illuminate the tympanum. The membrane is divided at its margin close to the bone all the way round. This is done with a blunt-pointed myringotome. The malleus is then seized at the upper end above the processus brevis with a pair of small strong forceps, such as Hartman's, and removed by applying traction downwards and then outwards. Provided there are no dense adhesions and no caries

Fig. 1.—A Modified Attic Punch Forceps.

of its neck at or above the part grasped in the forceps, the ossicle is satisfactorily extracted in this way. Removal of the incus is more difficult, and yet very important, for it is the incus which is more frequently carious, obstructs the aditus, and hinders free drainage from the antrum. To make more certain of the removal of this ossicle it is best to cut away the upper part of the margo tympanicus, either with a small, strong, cross-handled curette, or with such an instrument as Faricci's "pinza osteotoma." If the latter is chosen it is passed down the meatus with the hook-like plate of the "male" part upwards into the attic, into the position previously occupied by the head of the malleus. The cutting edge of the female part is forced down, and the bony lamella of the upper tympanic margin cut away, with some five to ten cutting manipulations. Care is necessary to avoid pressure on the inner tympanic wall and facial canal. As soon as the outer attic wall has been sufficiently removed the incus must be sought for. To do this a curved blunt incus hook, or curved ring, is directed with the point upwards in the position occupied by the malleus, and is then turned back, and with a rotatory movement the incus is drawn from the aditus downwards and forwards into the tympanum, whence it can be removed by forceps or syringing. Considerable care is to be taken lest the incus be displaced back into the antrum, both while attempting its extraction, and previously while removing the outer attic wall. Some operators prefer to attempt extraction of the incus before cutting away the upper and posterior part of the margo. Should the incus not be found it may perhaps be obtained by the antral syringe, or may be ensnared with loop of wire, but intra-aural extraction is, however, as a rule impossible, and the radical operation of opening the antrum is certainly necessary.

In curetting the granulations the position of the facial nerve must be remembered, in case the walls of the canal are carious and the nerve exposed. The stapes must also be left undisturbed in all intrameatal operations, for fear of setting up labyrinthitis. Other incidents arising from the operation are dizziness, which may last a few days; headache, usually quite transitory; and vomiting. Facial paralysis may come on, even though the nerve is uninjured, and is as a rule of short duration. The paralysis in such cases may be due to inflammatory compression, or hæmorrhage, or interference with the blood supply, or to other causes apart from laceration; direct injury to the nerve would be deplorable. Cases are recorded in which the jugular bulb has been wounded through the floor of the tympanum. Should this occur the hæmorrhage can be controlled by packing with gauze strips. The real danger is from infective thrombosis. Fortunately the conditions in which this accident is possible are rare.

Results.—Favourable results of ossiculectomy are sometimes quite striking, but it is never possible to predict with certainty the cases which will be followed by early cessation of discharge. Frequently suppuration continues uninterruptedly after extraction of the ossicles; diseases of other parts of the temporal bone generally co-exist in such cases. Its application is strictly limited to those cases of chronic suppurative otitis media in which there is no direct symptomatic evidence of disease of the accessory cavities of the tympanum, or of the facial nerve, labyrinth, or cranial contents.

On the whole the success of the procedure is only partial, and many otologists have given up its practice in favour of operations on the accessory cavities of the ear. There are, however, some distinct advantages which ossiculectomy possesses. It can be performed on patients who are unable to sacrifice three or four weeks at least from their duties for the performance of the radical operation, for after ossiculectomy the patient need only lie up a few days. Secondly, ossiculectomy is occasionally curative. Thirdly, the general condition of the patient with a suppurating ear is certainly benefited by the improvement in drainage thereby established. Fourthly, the restoration of hearing though not complete is often very considerable. Lastly, should perfect success not be obtained, it is a good preliminary to the radical operation.

*(To be continued.)*

## Figures and Tables

**Fig. 1. f1:**